# Electrophysiological Responses of Seleniferous Tea Seedlings to Cadmium Stress in *Astragalus sinicus*-Modified Substrates

**DOI:** 10.3390/plants15121897

**Published:** 2026-06-18

**Authors:** Jing Fan, Kun Zhai, Antong Xia, Dongshan Xiang, Haitao Yao, Xiangyong Gu, Jiqian Xiang

**Affiliations:** 1 Hubei Key Laboratory of Selenium Resource Research and Biological Application, Hubei Minzu University, Enshi 445000, China; 202430385@hbmzu.edu.cn (J.F.); zk3100@sohu.com (D.X.); 202330371@hbmzu.edu.cn (X.G.); 2 School of Chemical and Environmental Engineering, Hubei Minzu University, Enshi 445000, China; 3 Enshi Dachong Agricultural Technology Co., Ltd., Enshi 445000, China; yaoahaitao@163.com; 4 Enshi Tujia and Miao Autonomous Prefecture Academy of Agricultural Sciences, Enshi 445000, China

**Keywords:** electrophysiological monitoring, cadmium stress, phytoremediation, metabolic activity, in situ monitoring, smart agriculture

## Abstract

Seleniferous tea seedlings from Enshi, China, face cadmium (Cd) contamination risks due to the co-occurrence of selenium and cadmium in local soils, posing food safety concerns. While *Astragalus sinicus*-modified substrates are commonly applied for cadmium remediation, the performance of different monitoring techniques remains inadequately evaluated. This study compared four monitoring methods—growth traits, photosynthesis, chemical Cd removal rate, and plant electrophysiological parameters—in a pot experiment under cadmium stress (10 mg/kg Cd^2+^). Two tea varieties, Longjing 43 (*Camellia sinensis* ‘Longjing 43’. LJ 43) and Yulu 1 (*Camellia sinensis* ‘Yulu 43’. YL 1), were treated with four modified substrates (M1–M4). Specifically, compared to the control (M1), LM3 increased metabolic activity (MA), electrical impedance (EGC), and electrochemical response (ECR) by 140.27%, 122.5%, and 124.41%, respectively. These increases were significantly greater than those observed for the conventional metrics: 52.70% in total biomass (TB), 109.31% in photosynthetic rate (Pn), and 64.15% in chemical Cd removal (R_Cd_). Similarly, in the YM4 treatment, MA and EGC increased by 214.91% and 178.66%, respectively, which also significantly exceeded the increments in TB (48.74%), Pn (116.19%), and R_Cd_ (75.26%). Among the electrophysiological parameters, MA proved to be the most sensitive indicator, showing a strong correlation with Cd removal capacity. In conclusion, plant electrophysiology enabled real-time, in situ monitoring of cadmium remediation efficiency, offering a novel technological pathway to ensure the safety of seleniferous tea seedlings and advance precision agriculture.

## 1. Introduction

Enshi (E 109°04′–109°58′, N 29°50′–30°39′), known as the ‘World Selenium Capital,’ is home to the typical selenium-rich tea varieties Longjing 43 (*Camellia sinensis* ‘Longjing 43’, LJ 43) and Yulu 1 (*Camellia sinensis* ‘Yulu 43’, YL 1). Seleniferous tea seedlings have significant economic and health value [1,2,3,4]. However, the selenium-rich soil in Enshi is associated with high cadmium levels (0.78 mg/kg), with cadmium pollution being the most prevalent and severe issue [5,6], far exceeding the national safety standard for cadmium (0.097 mg/kg) [7]. Additionally, the cadmium (Cd) exceedance rate in Enshi soil reaches as high as 79.49% [8], posing a serious threat to the growth of seleniferous tea seedlings and the quality production of seleniferous tea. It is noteworthy that cadmium accumulates in the human body through the food chain, causing damage to kidney and bone health [9,10,11]. Implementing cadmium reduction measures for seleniferous tea seedlings is of significant importance for ensuring the food safety of seleniferous tea seedlings.

To address cadmium contamination in agricultural products, a variety of physical, chemical, and agronomic regulation techniques have been developed to ensure safety. Among these, the application of exogenous nutrients (e.g., selenium, silicon, and boron) represents an important strategy, as they can effectively reduce cadmium accumulation in crops by modulating plant physiological metabolism [12]. However, such methods mainly focus on lowering cadmium accumulation by influencing the plant’s own physiological processes, while techniques for the rapid and in situ monitoring and evaluation of the cadmium removal efficiency of remediation materials (e.g., soil amendments) themselves are still notably lacking.

The modified matrix of *Astragalus sinicus* is a typical cadmium-reducing material, which has the advantages of environmental friendliness and easy access compared to physical and chemical methods [13,14]. Recent studies have developed *Astragalus sinicus* L. biomass into engineered adsorbents, such as sulfur-modified biochar, through treatment with agents like Na_2_S, (NH_4_)_2_HPO_4_, and CaO, followed by pyrolysis. These functionalized materials can effectively immobilize cadmium ions via adsorption and precipitation, thereby reducing cadmium bioavailability in soil and alleviating cadmium stress in plants [15,16]. Previous studies have focused on the effects of *Astragalus sinicus*-modified substrates on plant cadmium absorption and transport. However, there is a lack of research into the cadmium removal efficiency of *Astragalus sinicus*-modified substrates. Exploring the impact of *Astragalus sinicus*-modified substrates on cadmium removal efficiency in seleniferous tea seedlings could facilitate more efficient cadmium reduction.

With the development of smart agriculture, in situ monitoring of crop life status has gradually emerged. In previous studies, invasive monitoring techniques that damage plant tissues and cells, such as ICP-MS, were commonly employed. These techniques were combined with growth parameters, such as root, stem, and leaf biomass, to quantitatively analyze differences in cadmium transport among organs [17]. Additionally, photosynthesis, including net photosynthetic rate (Pn) and transpiration (Tr), serves as a critical indicator for describing the cadmium removal efficiency of various environmental materials [18]. However, the aforementioned methods not only fail to achieve in situ monitoring of cadmium removal efficiency but are also limited by non-physiological factors such as chemical reagents [19].

In contrast, plant electrophysiology technology offers the advantages of non-destructiveness and real-time monitoring and has been widely applied in the in situ monitoring of crop growth dynamics. Based on plant electrophysiological techniques, Xiao investigated the electrophysiological characteristics of water metabolism and nutrient transport, revealing that bicarbonate and selenium interact to modulate cadmium transport, with low levels of bicarbonate enhancing selenium’s effect in reducing cadmium translocation in *L. chinensis* [20]. Xia investigated electrophysiological active transport metabolism, elucidating that a specific combination of bicarbonate and selenium can synergistically alleviate cadmium stress by promoting nutrient translocation efficiency and inhibiting cadmium uptake in *Euphorbia humifusa* [21]. Zhang measured electrophysiological water use efficiency and nutrient transport flux, demonstrating that these electrical parameters can effectively reflect changes in membrane protein composition and nutrient transport strategies in *Nerium oleander* and *Brassica napus* under biotic stress [22]; Wei measured the intrinsic capacitance and electrophysiological metabolic activity of tea plant leaves, showing that electrical characteristics can quickly and sensitively indicate the response of plant water metabolism to environmental drought stress [23]. However, the electrophysiological characteristics specifically related to cadmium removal efficiency in tea seedlings remain to be fully evaluated.

This study conducted pot experiments simulating cadmium stress, selecting two seleniferous tea seedlings (LJ 43 and YL 1, Enshi) and four *Astragalus sinicus*-modified substrates (M1–M4). By comparing four monitoring methods—growth monitoring, photosynthesis, chemical cadmium removal efficiency, and electrophysiological cadmium removal characteristics—the following objectives were addressed: (1) evaluate the sensitivity and potential of plant electrophysiological parameters as non-invasive indicators for monitoring cadmium removal efficiency by comparing their responses with conventional methods; (2) investigate the differences in electrophysiological cadmium removal efficiency of *Astragalus sinicus*-modified substrate on different seleniferous tea seedlings; and (3) screen optimal electrophysiological cadmium removal parameters, providing reliable smart agriculture technology for the food safety of seleniferous tea seedlings.

## 2. Materials and Methods

### 2.1. Experimental Treatment

The seleniferous tea seedlings (*Camellia sinensis*) used in this study were annual cuttings, varieties Longjing 43 (*Camellia sinensis* ‘Longjing 43’. LJ 43) and Yulu 1 (*Camellia sinensis* ‘Yulu 43’. YL 1), provided by the Agricultural Academy of Enshi Tujia and Miao Autonomous Prefecture (E 109°51′, N 30°33′). From May to July 2024, pot experiments were conducted at the Engineering Experimental Training Center of Hubei Minzu University (E 109°29′, N 30°18′). The substrate was a mixture of perlite and vermiculite (volume ratio 1:1). Healthy tea seedlings with uniform growth were selected, rinsed with distilled water three times to clean the roots, and transplanted into plastic pots (diameter 14.5 cm, height 11.5 cm), with 4 plants per pot and 4 pots per treatment. Subsequently, seedlings were acclimated for 7 days using 1/2 Hoagland nutrient solution (Qingdao, China). The indoor culture conditions were maintained as follows: daily irrigation with 100 mL of half-strength Hoagland solution, a temperature of 25 °C, relative humidity of 75%, and a 12 h photoperiod (6:00 a.m. to 6:00 p.m.) under LED lights with a photosynthetic photon flux density (PPFD) of 300 µmol/(m^2^·s) [24]. The *Astragalus sinicus*-modified substrate (adsorbent) was prepared according to methods described in references [25,26] with slight modifications. Table 1 shows the setup of the experimental group. After the 7-day acclimatization period, cadmium (Cd) stress treatment was initiated. Cadmium chloride (CdCl_2_, analytical grade, purity ≥ 99%, Shanghai Macklin Biochemical Technology Co., Ltd., Shanghai, China) was used as the cadmium source and was added exogenously at a concentration of 10 mg/kg to simulate cadmium contamination levels typical of Enshi farmland soils [8]. The cadmium stress treatment, applied as a CdCl_2_ solution, lasted for 60 days. Simultaneously, the pre-weighed *Astragalus sinicus*-modified substrate (treatments M1–M4) was thoroughly and uniformly incorporated into the perlite–vermiculite growth medium as an adsorbent. This ensured even distribution of the adsorbent in the rhizosphere, enabling interaction with Cd^2+^ ions from the onset of stress. The control (CK) received only CdCl_2_ solution without the adsorbent. Throughout the 60-day period, the aforementioned growth conditions were maintained, and the nutrient solution was replenished regularly.

### 2.2. Growth Characteristics

#### 2.2.1. Biomass of Roots, Stems, and Leaves

After harvesting, the roots, stems, and leaves of the tea seedlings were washed and weighed using a precision balance with a resolution of 0.0001 g (AR124CN, Shanghai Aohaus Instrument Co., Ltd., Shanghai, China) (*n* = 4).

#### 2.2.2. Chlorophyll and Total Nitrogen Content

The total chlorophyll and total nitrogen content in leaves were measured using a chlorophyll meter (TYS-4N, Beijing Jinke Lida Electronic Technology Co., Ltd., Beijing, China) (*n* = 4).

### 2.3. Photosynthesis

From 9:00 A.M. to 11:00 A.M., an LI-6400 portable photosynthetic instrument (LI-COR, Beijing Ligao Tai Technology Co., Ltd., Beijing, China) was used to measure photosynthesis on the second or third fully expanded leaf. The measurements included: net photosynthetic rate (Pn, µmol·m^−2^·s^−1^), stomatal conductance (Gs, mol·m^−2^·s^−1^), intercellular CO_2_ concentration (Ci, μmol·mol^−1^), and transpiration rate (E, mmol·m^−2^·s^−1^). The experimental conditions were: light intensity of 1000 μmol·m^−2^·s^−1^, air flow rate of 700 μmol·s^−1^, leaf temperature of 25 °C, relative humidity of 60%, and atmospheric CO_2_ concentration of 400 μmol·mol^−1^.

### 2.4. Cadmium Enrichment-Transport Characteristics and Cadmium Removal Efficiency

The tea seedling roots, stems, and leaves were heated at 105 °C for 30 min, then dried at 75 °C until a constant weight was achieved. The dried tea seedling samples were ground using a grinder and sieved through a 100-mesh sieve. After digestion, the total cadmium content in each tissue of the tea seedling roots, stems, and leaves was determined using inductively coupled plasma mass spectrometry (ICP-MS, Thermo Fisher Scientific, Waltham, MA, USA) [27]. The measurement results were validated using the following quality parameters: linearity (R^2^ > 0.999), detection limit of 0.05 μg/L, quantification limit of 0.15 μg/L, and repeatability (RSD < 5%). Subsequently, the bioconcentration factor (BCF_Cd_), translocation factor (TF_Cd_), and chemical removal efficiency (R_Cd_) of cadmium in tea seedlings were calculated using formulas from previous studies, as shown in Formulas (1)–(3).

(1) $${\mathrm{BCF}}_{\mathrm{Cd}}=\frac{C_{Cd-shoot}}{C_{Cd-soil}}$$

(2) $${\mathrm{TF}}_{\mathrm{Cd}}=\frac{C_{Cd-shoot}}{C_{Cd-root}}$$

(3) $$R_{Cd}(\%)=\frac{{\left(CK-EG\right)}_{C_{Cd-shoot}}}{{CK}_{C_{Cd-root}}}\times 100$$

“CK” denotes the control group (which did not receive the adsorbent treatment) and “EG” denotes the experimental group (which received the *Astragalus sinicus*-modified substrate treatment).

### 2.5. Electrophysiological Cadmium Removal Efficiency

In this study, the electrophysiological cadmium removal efficiency was quantified through three parameters: electrophysiological relative metabolic activity (MA), electrophysiological growth capacity (EGC), and electrophysiological comprehensive cadmium resistance (ECR). The derivation steps are as follows.

#### 2.5.1. Intrinsic Electrophysiological Characteristics of Leaves

**Figure 1:**
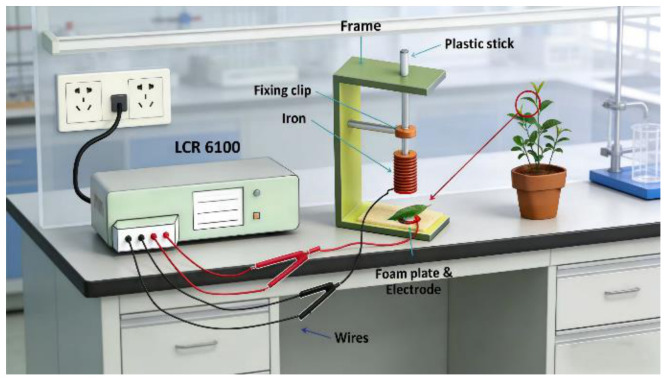
General view of the plant electrophysiological measurement setup.

A schematic representation of the plant electrophysiology measurement system is provided in Figure 1. The system included a self-assembled plant electrode interface device connected to an LCR meter (LCR-6100, Good-Ark Electronics Co., Ltd., Suzhou, China). The measurement voltage and frequency were set to 1.5 V and 3 kHz, respectively. Well-growing and uniformly developed tea seedlings were selected, with four seedlings measured per group. The second fully unfolded leaf from the top was used for measurement. After removing dust and moisture from the leaf surface, the leaf was placed between parallel electrode plates for electrophysiological recording.

Following the method described by Xiao [21], different electrophysiological clamping forces (F = 1–7 N, with an increment of 1 N per measurement) were applied. Leaf capacitance (Cp), resistance (R), and impedance (Z) were measured at each clamping force. Physiological capacitive reactance (Xc) and inductive reactance (X_L_) were subsequently calculated. Electrophysiological equations (Formulas (4)–(8)) were established to describe the relationships between the five electrical parameters and F.

(4) $$Cp=y_{0}+a\;F$$

where F is the clamping force; y0-F is the initial capacitance value when F is zero (i.e., the intercept); a: the change in leaf capacitance per unit force F (i.e., the slope).

(5) $$R=y_{1}+a_{1}e^{-b_{1}F}$$

where y_1_ is the minimum resistance, i.e., the theoretical minimum value (i.e., intercept) of resistance convergence when F approaches infinity; a_1_ is the initial resistance variation amplitude; and b1 is the decay constant.

(6) $$Z=y_{2}+a_{2}e^{{-b}_{2}F}$$

(7) $$Xc=y_{3}+a_{3}e^{{-b}_{3}F}$$

(8) $$X_{L}=y_{4}+a_{4}e^{{-b}_{4}F}$$

When F = 0, the intrinsic electrophysiological parameters of the leaf were derived, including: intrinsic capacitance (ICp), intrinsic resistance (IR), intrinsic impedance (IZ), intrinsic capacitive reactance (IXc), and intrinsic inductive reactance (IX_L_), as presented in Formulas (9)–(13).

(9) $$IR=y_{1}+a_{1}$$

(10) $$IZ=y_{2}+a_{2}$$

(11) $$IXc=y_{3}+a_{3}$$

(12) $$IX_{L}=y_{4}+a_{4}$$

(13) $$ICp=\frac{1}{2\pi f\;IXc}$$

where f is the frequency.

#### 2.5.2. Electrophysiological Characteristics of Intracellular Water Metabolism and Nutrient Transport in Leaves

The electrophysiological characteristics associated with intracellular water metabolism and nutrient transport in plant leaves were calculated according to the method described in reference [28]. These parameters included: intracellular water holding capacity (IWHC), intracellular water holding time (IWHT), intracellular water transfer rate (WTR), unit nutrient flux (UNF), active nutrient transport flux (UAF), and active nutrient transport capacity (NAC), as detailed in Formulas (14)–(19).

(14) $$IWHC=\sqrt{{\left(ICp\right)}^{3}}$$

(15) IWHT=ICp×IZ

(16) $$WTR=\frac{IWHC}{IWHT}$$

(17) $$UNF=\frac{R}{IXc}+\frac{R}{{IX}_{L}}$$

(18) $$UAF=\frac{IXc}{IX_{L}}$$

(19) NAC=UAF×WTR

#### 2.5.3. Electrophysiological Metabolic Characteristics and Growth Viability

Electrophysiological metabolic characteristics and growth vigor were calculated [29]. Nutrient translocation capacity (NTC) is equal to UNF multiplied by WTR. The leaf electrophysiological metabolic characteristics include: relative metabolic flux (MF), relative metabolic rate (MR), relative metabolic activity (MA), and electrophysiological growth capacity (EGC), as shown in Formulas (20)–(23).

(20) $$MF=\frac{10^{5}}{IR\times IZ\times IXc\times IX_{L}}$$

(21) MR=WTR×NAC

(22) $$MA=\sqrt[{6}]{MF\times MR}$$

(23) $$EGC=\sqrt[{4}]{IWHC\times IWHT\times NTC\times MA}$$

#### 2.5.4. Electrophysiological Cadmium Resistance

The electrophysiological cadmium transport characteristics of tea seedling leaves [30] were calculated, including leaf electrophysiological cadmium transport capacity (STC_Cd_). Based on cadmium excretion capacity (EC1), cell wall thickness (d), dilution capacity (EC2), and ultrafiltration capacity (EC3), the electrophysiological comprehensive cadmium resistance (ECR) was calculated, as shown in Formulas (24)–(29).

(24) $${STC}_{Cd}=UNF\times WTR$$

(25) $$EC1=0.25\times (IR+IZ+IXc+{IX}_{L})$$

(26) $$d=\frac{U^{2}a}{2}$$

where U is the applied test voltage (3 kHz).

(27) $$EC2=0.25\times (ICp+d+IWHC+{STC}_{Cd})$$

(28) EC3=0.25×(IWUE+IWHT+UNF+WTR)

where IWUE is calculated as d divided by IWHC.

(29) $$ECR=\frac{EC1+EC2+EC3}{3}$$

### 2.6. Data Processing

Statistical analysis was performed using WPS Office 2021 software; SigmaPlot Data 12.0 was employed to fit plant electrophysiological equations; SPSS 20.0 was used for one-way analysis of variance (ANOVA), and multiple comparisons were conducted using the Waller-Duncan test (*p* < 0.05); Origin 2024 was used for data visualization. All experimental treatments were set with *n* = 4, and data were expressed as “mean ± standard deviation”.

## 3. Results

### 3.1. Effects on Growth Characteristics

Table 2 presents the effects of different *Astragalus sinicus*-modified substrates on the biomass of various organs in two seleniferous tea seedlings. The results showed that the total biomass of LM2, LM3, and LM4 increased by 25.00%, 52.70%, and 18.24%, respectively, compared to LM1 (control). The total biomass of YM2, YM3, and YM4 increased by 19.86%, 34.66%, and 48.74%, respectively, compared to YM1 (control). The biomass of roots, stems, and leaves was the highest in LM3 and YM4, with LM3 showing increases of 58.19%, 105.00%, and 32.32% compared to LM1 (control), while YM4 showed increases of 32.34%, 72.22%, and 76.71% compared to YM1. Table 3 demonstrates the effects of different *Astragalus sinicus*-modified substrates on the total chlorophyll content and total nitrogen content in the two tea seedlings. The results indicate that LM3 achieved the highest total chlorophyll and total nitrogen content, increasing by 43.00% and 43.70%, respectively, compared to LM1. YM4 also showed the highest total chlorophyll content and total nitrogen content, increasing by 37.71% and 25.14%, respectively, compared to YM1. There were no significant differences in total chlorophyll content and total nitrogen content between LM2 and LM4, but both were significantly higher than LM1.

### 3.2. Effects on Photosynthesis

**Figure 2:**
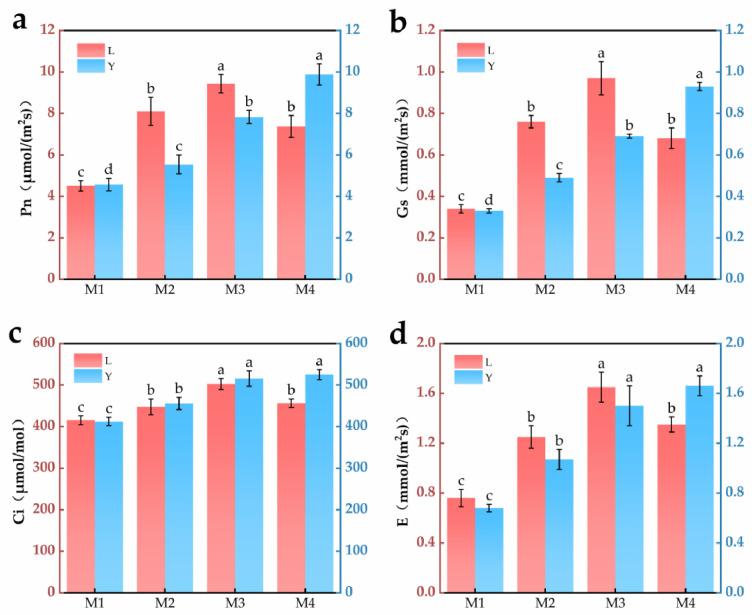
Photosynthetic characteristics of tea leaves under different treatments: (**a**) net photosynthetic rate, Pn; (**b**) stomatal conductance, Gs; (**c**) intercellular CO_2_ concentration, Ci; (**d**) transpiration rate, E. Different lowercase letters above the bars indicate significant differences at *p* < 0.05.

Figure 2 illustrates the effects of different *Astragalus sinicus*-modified substrates on photosynthesis in two seleniferous tea seedlings. The values of Pn, Gs, Ci, and E in LM1–LM4 initially increased, then declined, whereas YM1–YM4 showed sustained growth. The Pn, Gs, Ci, and E in LM2, LM3, and LM4 were significantly higher than those in LM1, with LM3 recording the highest values at 9.44 µmol·m^−2^·s^−1^ for Pn, 0.97 mmol·m^−2^·s^−1^ for Gs, 502.56 μmol·mol^−1^ for Ci, and 1.65 mmol·m^−2^·s^−1^ for E. LM2 and LM4 followed closely without significant differences. Overall, the photosynthetic rates in YM2, YM3, and YM4 were significantly higher than those in YM1, with YM4 showing the maximum increase. The Pn, Gs, Ci, and E in YM4 were 1.16-fold, 1.82-fold, 0.27-fold, and 1.44-fold higher than those in YM1, respectively.

### 3.3. Cadmium Enrichment/Transport Coefficients and Chemical Cadmium Removal Efficiency

Table 4 demonstrates the effects of different *Astragalus sinicus*-modified substrates on the bioconcentration coefficient (BCF_Cd_), transfer coefficient (TF_Cd_), and chemical cadmium removal rate (R_Cd_) of two tea seedlings. The BCF_Cd_ and TF_Cd_ of LM1 to LM4 exhibited a pattern of initial decline followed by recovery, with LM3 showing the lowest values at 64.15% and 36.90% lower than LM1, respectively. YM1 to YM4 demonstrated a continuous decrease in BCF_Cd_ while TF_Cd_ initially increased before declining. YM4 recorded the lowest BCF_Cd_ and TF_Cd_, 75.00% and 46.15% lower than YM1, respectively. Both LM3 and YM4 achieved the highest R_Cd_, reaching 64.15% and 75.26%, respectively.

### 3.4. Intrinsic Electrophysiological Parameters of the Leaf

**Figure 3:**
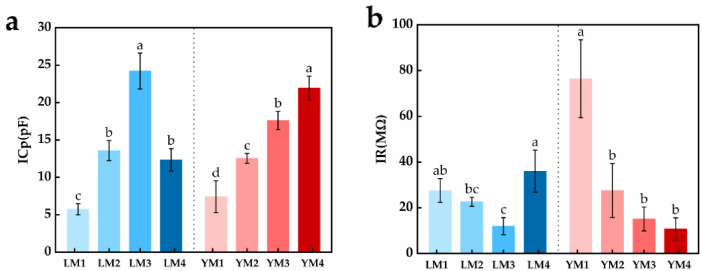

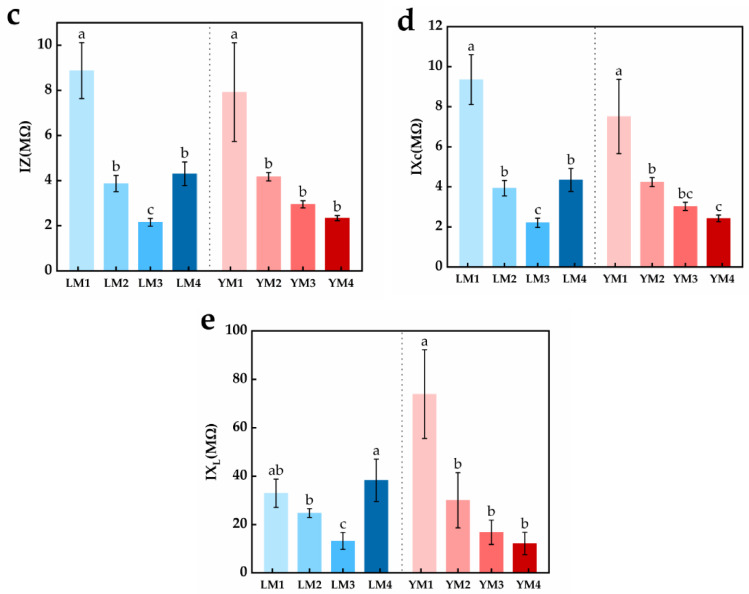
Electrophysiological parameters of tea leaves under different treatments: (**a**) inherent capacitance, ICp; (**b**) inherent resistance, IR; (**c**) inherent impedance, IZ; (**d**) inherent capacitive reactance, IXc; (**e**) inherent inductive reactance, IX_L_. Different lowercase letters above the bars indicate significant differences at *p* < 0.05. The dotted line separates the LM group (LM1–LM4) from the YM group (YM1–YM4).

Figure 3 illustrates the effects of different *Astragalus sinicus*-modified substrates on the intrinsic electrophysiological parameters of leaves from two seleniferous tea seedlings. LM3 exhibited the highest ICp, which was 3.22 times higher than that of LM1. The IR, IZ, IXc, and IX_L_ of LM3 were reduced by 56.47%, 75.74%, 76.42%, and 60.00%, respectively, compared to LM1. YM4 showed the highest ICp, which was 1.96 times higher than that of YM1. The IR, IZ, IXc, and IX_L_ of YM4 were decreased by 71.69%, 70.44%, 67.74%, and 83.60%, respectively, compared to YM1.

### 3.5. Effects on Electrophysiological Characteristics of Intracellular Water Metabolism and Nutrient Transport in Leaves

Table 5 presents the effects of different *Astragalus sinicus*-modified substrates on electrophysiological water metabolism and nutrient transport in two seleniferous tea seedlings. In electrophysiological water metabolism, LM3 and YM4 exhibited the highest IWHC, IWUE, and WTR values. Compared to LM1, LM3 showed increases of 161.30%, 76.61%, and 153.10% in IWHC, IWUE, and WTR, respectively. YM4 demonstrated significant increases of 107.33%, 53.36%, and 123.72% in IWHC, IWUE, and WTR compared to YM1. For electrophysiological nutrient transport, LM4 achieved the highest UNF value, showing a 152.66% increase over the control LM1. No significant changes were observed in UNF and UAF for YM1 to YM4. Both LM3 and YM4 exhibited the highest NAC values, with increases of 58.24% and 76.32%, respectively, compared to M1 (control).

### 3.6. Electrophysiological Metabolic Characteristics and Growth Vigor of Leaves

**Figure 4:**
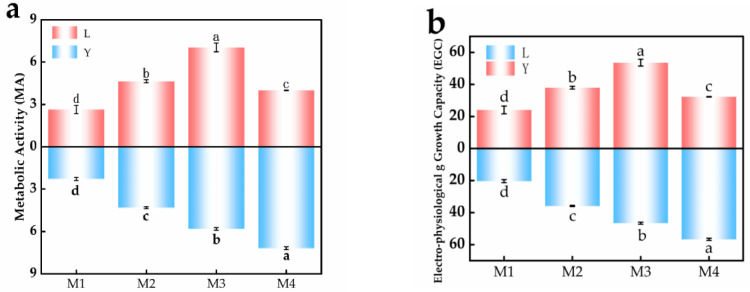
Metabolic activity and cadmium resistance of tea seedling leaves: (**a**) leaf metabolic activity, LMA; (**b**) leaf growth resistance, EGC. Different lowercase letters above the bars indicate significant differences at *p* < 0.05.

Figure 4 illustrates the effects of different *Astragalus sinicus*-modified substrates on the electrophysiological metabolic characteristics and growth vigor of two seleniferous tea seedlings. The MA and EGC of LM3 and YM4 were the highest, with LM3 increasing by 140.27% and 122.50% compared to LM1, respectively, and YM4 increasing by 214.91% and 178.66% compared to YM1, respectively.

### 3.7. Cadmium Resistance in Leaf Electrophysiology

**Figure 5:**
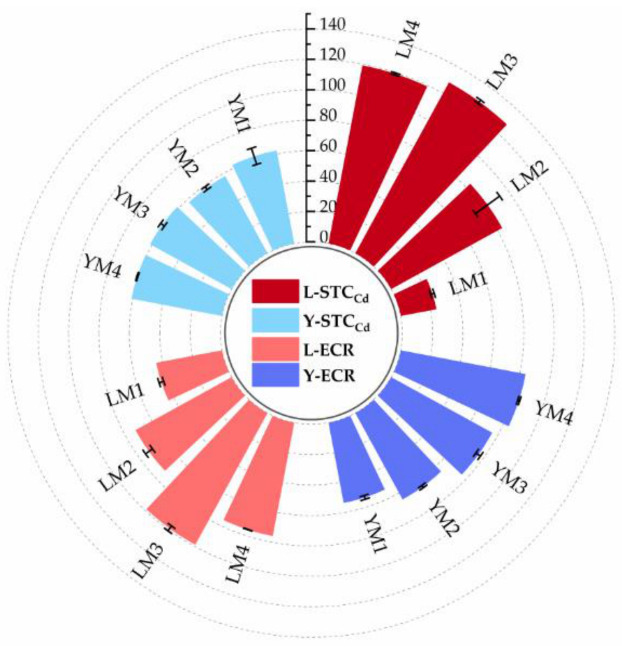
Electrophysiological characteristics of cadmium transport in tea seedling leaves.

Figure 5 illustrates the effects of different *Astragalus sinicus*-modified substrates on the electrophysiological cadmium transport capacity (STC_Cd_) and electrophysiological cadmium tolerance (ECR) of two seleniferous tea seedlings. LM3 exhibited the highest STC_Cd_ and ECR, increasing by 436.81% and 124.41%, respectively, compared to LM1. YM4 showed no significant change in STC_Cd_ but demonstrated the highest ECR, increasing by 55.94% over YM1.

### 3.8. Principal Component Analysis

**Figure 6:**
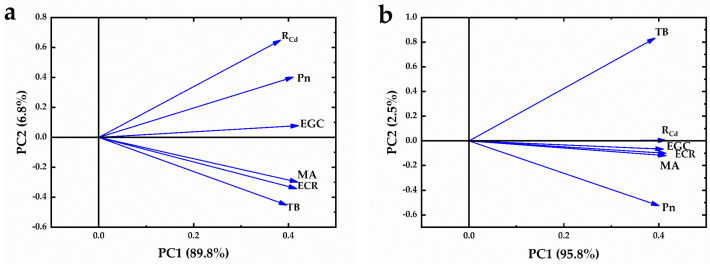

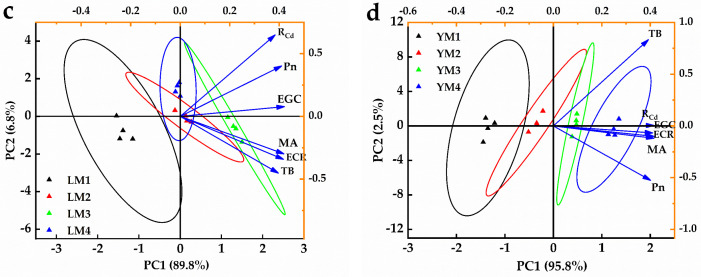
Principal component analysis (PCA) of two seleniferous tea seedlings: (**a**) Loading plot of LJ43; (**b**) Loading plot of YL1; (**c**) Biplot of LJ43; (**d**) Biplot of YL1.

Figure 6 presents the principal component analysis (PCA) based on the two seleniferous tea seedlings’ traits of TB, Pn, R_Cd_, and electrophysiological cadmium removal characteristics (MA, EGC, and ECR). Among the PCA scores, PC1 accounted for the largest portion of each factor’s variance. For LM1–LM4, PC1 and PC2 accounted for 89.8% and 6.8% (Figure 6a), respectively, while for YM1–YM4, PC1 and PC2 accounted for 95.8% and 2.5% (Figure 6b). LJ43 exhibited higher PC1 scores for MA (0.42), EGC (0.42), and ECR (0.42) compared to TB (0.40), Pn (0.41), and R_Cd_ (0.38). Similarly, YL1 demonstrated higher PC1 scores for MA (0.42), EGC (0.42), and ECR (0.41) than TB (0.39), Pn (0.40), and R_Cd_ (0.41). The PCA clustering results indicated that LM3 (Figure 6c) and YM4 (Figure 6d) achieved the highest PC1 scores.

## 4. Discussion

### 4.1. Sensitivity and Correlation of Plant Electrophysiological Indices with Cadmium Remediation

This study evaluated the response of the tea plant to cadmium-contaminated soils grown on different *Astragalus sinicus*-modified substrates. Consistent with previous reports showing a “low-concentration stimulation–high-concentration inhibition” pattern of photosynthetic parameters under Cd stress [31], the plant TB, Pn, and ICp of both tea cultivars exhibited a consistent trend of initial increase followed by a decrease across all treatments. Previous studies indicated that Cd stress can alter plant cell membrane permeability and induce regular changes in root dielectric properties (capacitance and impedance), with decreased capacitance generally reflecting impaired membrane integrity and suppressed metabolic activity [32,33]. The electrophysiological results obtained in this study aligned with these findings, as higher Cd removal efficiency corresponded to more active mesophyll cell metabolism, which was reflected by higher physiological capacitance (ICp) and lower physiological resistance (IR, IZ, IXc, IX_L_). The synchronous variation among growth indices, photosynthetic efficiency, and electrophysiological parameters (ICp) further supported the reliability of electrophysiological methods as indicators of plant stress responses. The data showed that the LM3 and YM4 treatments were the most effective Cd removal strategies for Longjing 43 and Yulu 1, respectively, resulting in the greatest increases in TB, Pn, and ICp. PCA revealed close correlations among the monitoring indicators, demonstrating the high sensitivity of electrophysiological parameters in distinguishing the effects of different substrate amendments. Specifically, under optimal treatment conditions, the relative changes in electrophysiological indicators (MA, EGC, and ECR) were significantly greater than those of TB (Table 1), Pn (Figure 2), and R_Cd_ (Table 4) relative to the control group. Although this suggests the high sensitivity of the method, the present study did not propose that electrophysiological indicators are universally superior to conventional physiological detection methods; the PCA results merely reflected the unique response characteristics of the electrophysiological system. Electrophysiological approaches should not be considered alternatives but rather valuable complementary tools. Their in situ, real-time, and non-destructive detection features provide distinct advantages for capturing dynamic physiological changes associated with Cd removal efficiency, which aligns with recent perspectives on the use of plant impedance/capacitance methods for in situ monitoring of heavy metal stress responses [34,35]. The significant correlations observed between electrophysiological indicators and photosynthetic and growth parameters further demonstrated the potential of specific electrophysiological parameters as sensitive proxies for evaluating the efficacy of soil amendments, though validation with larger sample sizes is needed. In summary, this study confirmed that plant electrophysiological indicators can sensitively characterize tea plant responses to Cd-contaminated substrate amendments and Cd removal efficiency, providing strong evidence for the application of this technology as a novel non-destructive means for functional phenotyping in phytoremediation processes.

### 4.2. Effects of Different Modifying Substrates of Astragalus sinicus L. on Cadmium Removal Efficiency of Selenium-Rich Tea Seedlings by Electrophysiological Method

The differences in MA, EGC, and ECR of tea plants were attributed to their leaf electrophysiological water metabolism and nutrient transport characteristics. The maximum values of IWHC, IWUE, WTR, and NAC were observed in LM3 and YM4 (Table 5), indicating that under these conditions, the two seleniferous tea seedlings exhibited optimal water retention capacity, water use efficiency, and transport capacity. This enhanced their active nutrient transport ability [36], which was attributed to the maximum values of TB (Table 2) and Pn (Figure 3a) in LM3 and YM4, promoting MA and reducing cadmium stress in tea plants, thereby maximizing R_Cd_ (Table 4) and ECR (Figure 5) in LM3 and YM4. Additionally, the differences in cadmium removal efficiency between the two seleniferous tea seedlings were attributed to their varying selenium enrichment capacities [37]. Compared to LJ 43, YL 1 demonstrated a stronger selenium enrichment capacity. Studies have shown that under insufficient exogenous selenium supply, selenium-enriched plants preferentially utilize sulfate to antagonize cadmium, promoting CdS formation by upregulating SULTR expression [38], thereby reducing Cd^2+^ stress. In this study, the sulfate content of M4 was higher than that of M3 (Table 1). Considering that YL 1 exhibited stronger selenium enrichment capability compared to LJ 43, one possible explanation for the highest ECR in YM4 is that YL 1 might utilize more sulfate to antagonize Cd [39,40], resulting in the highest ECR of YM4. Conversely, LJ 43 demonstrated weaker selenium enrichment than YL 1, leading to inferior sulfate antagonism against cadmium, as reflected in the highest ECR in LM3. These interpretations, while consistent with the observed correlations, remain to be verified by direct mechanistic studies.

### 4.3. Sensitivity of Electrophysiological Metabolic Activity (MA) to Cadmium Remediation

The analysis of PCA loadings (Figure 6a,b) indicated that electrophysiological indices, including MA, EGC, and ECR, exhibited high sensitivity to treatment variations, with response magnitudes appearing more pronounced than those of TB, Pn, and R_Cd_. Additionally, PCA clustering (Figure 6c,d) demonstrated that these indices effectively differentiated cadmium removal efficiencies across various *Astragalus sinicus*-modified substrates, with distinct separation observed for LM3 and YM4. Among these indices, MA emerged as a particularly responsive parameter, showing marked increases under the optimal treatments (LM3 and YM4) compared to the control (M1). Further analysis suggested potential physiological associations with MA dynamics. WTR and NAC were identified as factors correlated with MA variation [41], indicating that *Astragalus sinicus* modifications may influence cadmium removal efficiency partly through modulating water transport and nutrient uptake capacities in the leaves of both cultivars [42,43]. For instance, YL 1, a seleniferous cultivar, is known for its enhanced sulfate transport capacity attributed to selenium-sulfur co-metabolism [44]. Consistent with this, YM4 exhibited superior photosynthesis and growth rates, which coincided with maximal MA values. Conversely, LJ 43, a non-seleniferous cultivar with relatively weaker sulfate transport [45], still showed that LM3 induced higher photosynthesis and growth than LM4, corresponding to the highest MA values in this cultivar. In conclusion, the strong correlation between MA and chemically measured cadmium removal efficiency suggests that MA serves as a sensitive electrophysiological proxy for assessing the efficacy of remediation substrates. While not a direct measure of Cd uptake, the non-destructive and dynamic nature of MA makes it a promising phenotypic indicator for screening substrate amendments, with higher values generally reflecting more vigorous plant physiological responses to effective Cd immobilization/removal.

## 5. Conclusions

This study evaluated the electrophysiological responses of two seleniferous tea seedlings to cadmium stress in *Astragalus sinicus* L.-modified substrates. The results indicated that treatments M3 and M4 achieved optimal cadmium removal ratios for cultivars LJ 43 and YL 1, respectively. Compared to conventional growth and photosynthetic parameters, electrophysiological indicators—particularly MA—exhibited more pronounced responses to variations in substrate remediation efficiency. The significant increments in MA, EGC, and ECR under effective treatments suggest that these parameters are highly sensitive to cadmium stress and substrate modification. Among the measured variables, MA demonstrated the strongest correlation with cadmium removal efficiency, highlighting its potential as a non-invasive proxy indicator for assessing remediation performance in seleniferous tea seedlings. While this pot-scale experiment demonstrated the feasibility and sensitivity of plant electrophysiological techniques for in situ monitoring, further field validation is necessary to confirm their robustness and predictive accuracy under complex agronomic conditions. Overall, this study positions plant electrophysiological monitoring as a valuable complementary approach to conventional chemical and physiological assessments, offering an innovative technological avenue for ensuring the safety of seleniferous tea seedlings and advancing precision agriculture.

## Figures and Tables

**Table 1:** Experimental design of the modified substrate of *Astragalus sinicus* L.

Classify	M1	M2	M3	M4
LJ 43 (L)	LM1	LM2	LM3	LM4
YL 1 (Y)	YM1	YM2	YM3	YM4

Note: The cadmium-reducing material composition in a pot of 400 g substrate soil consists of *Astragalus sinicus* L., Na_2_S, (NH_4_)_2_HPO_4_, and CaO (unit: g) in mass ratio. The treatment groups are matched as follows: M1 (0.00, 0.00, 0.00, 0.00); M2 (0.08, 0.02, 0.08, 0.02); M3 (0.08, 0.04, 0.06, 0.02); M4 (0.08, 0.06, 0.04, 0.02).

**Table 2:** Biomass of roots, stems, and leaves in tea seedlings.

Treatment	Root FW g/Plant	Stem FW g/Plant	Leaves FW g/Plant	Total Biomass FW g/Plant
LM1	1.77 ± 0.31 c	0.20 ± 0.04 b	0.99 ± 0.08 b	2.96 ± 0.43 c
LM2	2.25 ± 0.23 b	0.32 ± 0.07 a	1.12 ± 0.14 ab	3.70 ± 0.36 b
LM3	2.80 ± 0.18 a	0.41 ± 0.06 a	1.31 ± 0.21 a	4.52 ± 0.44 a
LM4	2.16 ± 0.17 bc	0.31 ± 0.05 ab	1.02 ± 0.07 b	3.50 ± 0.27 bc
YM1	1.67 ± 0.14 c	0.36 ± 0.07 c	0.73 ± 0.12 c	2.77 ± 0.30 c
YM2	1.86 ± 0.18 bc	0.45 ± 0.08 bc	1.01 ± 0.08 b	3.32 ± 0.34 bc
YM3	1.98 ± 0.12 ab	0.65 ± 0.08 a	1.10 ± 0.12 ab	3.73 ± 0.29 ab
YM4	2.21 ± 0.11 a	0.62 ± 0.13 ab	1.29 ± 0.13 a	4.12 ± 0.30 a

Note: Each value represents the mean ± standard deviation (*n* = 4). Different lowercase letters indicate significant differences between groups (*p* < 0.05).

**Table 3:** Chlorophyll and total nitrogen contents in tea seedling leaves.

Treatment	Chlorophyll (SPAD)	Total Nitrogen (mg/g)
LM1	34.56 ± 2.75 d	13.64 ± 0.55 c
LM2	41.28 ± 2.84 c	17.96 ± 0.21 b
LM3	49.42 ± 1.34 a	19.60 ± 0.41 a
LM4	44.80 ± 1.66 b	17.66 ± 0.40 b
YM1	34.42 ± 2.27 d	14.16 ± 0.39 d
YM2	39.22 ± 1.34 c	15.18 ± 0.31 c
YM3	42.94 ± 1.21 b	16.50 ± 0.32 b
YM4	47.40 ± 1.83 a	17.72 ± 0.41 a

Note: Each value represents the mean ± standard deviation (*n* = 4). Different lowercase letters indicate significant differences between groups (*p* < 0.05).

**Table 4:** Cadmium enrichment factor (BCF_Cd_), translocation factor (TF_Cd_), and chemical removal efficiency (R_Cd_).

Treatment	BCF_Cd_	TF_Cd_ (10^−1^)	R_Cd_ (%)
LM1	1.06 ± 0.02 a	0.84 ± 0.02 a	NA
LM2	0.69 ± 0 b	0.63 ± 0.01 b	34.77 ± 1.17 c
LM3	0.38 ± 0 d	0.53 ± 0.01 c	64.15 ± 0.92 a
LM4	0.46 ± 0.02 c	0.42 ± 0.02 d	56.98 ± 1.98 b
YM1	0.72 ± 0 a	0.26 ± 0 a	NA
YM2	0.50 ± 0.06 b	0.28 ± 0.01 a	31.19 ± 7.3 c
YM3	0.31 ± 0.01 c	0.20 ± 0.01 b	56.47 ± 1.13 b
YM4	0.18 ± 0 d	0.14 ± 0.01 c	75.26 ± 0.52 a

Note: Each value represents the mean ± standard deviation (*n* = 4). Different lowercase letters indicate significant differences between groups (*p* < 0.05). NA, not applicable.

**Table 5:** Intracellular water metabolism and nutrient transport characteristics of tea leaves.

Treatment	IWHC	IWUE	WTR	UNF	UAF	NAC
LM1	320.06 ± 28.03 c	28.47 ± 2.46 c	6.37 ± 0.60 c	3.76 ± 0.17 c	0.29 ± 0.01 a	1.82 ± 0.25 ab
LM2	568.67 ± 37.30 b	40.21 ± 3.45 b	10.89 ± 0.66 b	6.74 ± 1.10 ab	0.16 ± 0.03 b	1.74 ± 0.19 ab
LM3	836.31 ± 55.42 a	50.28 ± 0.88 a	16.12 ± 0.73 a	6.47 ± 2.16 ab	0.18 ± 0.08 b	2.88 ± 1.08 a
LM4	533.51 ± 44.11 b	37.97 ± 1.73 b	10.14 ± 0.75 b	9.50 ± 3.04 a	0.12 ± 0.05 b	1.20 ± 0.39 b
YM1	377.94 ± 71.23 d	31.69 ± 8.15 b	6.83 ± 1.55 d	12.13 ± 5.65 a	0.11 ± 0.05 a	1.90 ± 0.19 c
YM2	539.55 ± 19.00 c	44.59 ± 1.59 a	10.32 ± 0.27 c	7.51 ± 3.20 a	0.15 ± 0.05 a	1.59 ± 0.53 bc
YM3	676.38 ± 31.36 b	51.77 ± 3.53 a	13.05 ± 0.41 b	5.99 ± 2.16 a	0.19 ± 0.06 a	2.49 ± 0.71 ab
YM4	783.60 ± 37.40 a	48.60 ± 0.32 a	15.28 ± 0.38 a	5.43 ± 2.44 a	0.22 ± 0.08 a	3.35 ± 1.17 a

Note: IWHC, intracellular water holding capacity; IWUE, intracellular water holding time; WTR, water transfer rate; UNF, unit nutrient flux; UAF, active nutrient transport flux; NAC, active nutrient transport capacity. Each value represents the mean ± standard deviation (*n* = 4). Different lowercase letters indicate significant differences between groups (*p* < 0.05).

## Data Availability

Data are contained within the article.

## Abbreviations

The following abbreviations are used in this manuscript:

TB	Total biomass
Pn	Net photosynthetic rate
R_Cd_	Chemical Cd removal
Gs	Stomatal conductance
Ci	Intercellular CO_2_ concentration
WUE	Water use efficiency
TF_Cd_	Cd transport coefficient
BCF_Cd_	Cd concentration coefficient
Cp	Capacitance
R	Resistance
ICp	Capacitive reactance
IR	Intrinsic resistance
IZ	Intrinsic impedance
IX_C_	Intrinsic capacitive reactance
IX_L_	Intrinsic inductive reactance
IWHC	Intracellular water holding capacity
IWHT	Intracellular water holding time
WTR	Water transfer rate
UNF	Nutrient flux per unit area
UAF	Active transport flow of nutrient
NAC	Nutrient active translocation capacity
MA	Metabolic activity
EGC	Electrophysiological growth capacity
ECR	Electrophysiological comprehensive cadmium resistance
